# Strategies of Dyadic Coping and Self-Regulation in the Family Homes of Chronically Ill Persons: A Qualitative Research Study Using the Emotional Map of the Home Interview Method

**DOI:** 10.3389/fpsyg.2019.00403

**Published:** 2019-02-28

**Authors:** Viola Sallay, Tamás Martos, Sheryl L. Chatfield, Andrea Dúll

**Affiliations:** ^1^Institute of Psychology, University of Szeged, Szeged, Hungary; ^2^College of Public Health, Kent State University, Kent, OH, United States; ^3^Institute of Psychology, Eötvös Loránd University, Budapest, Hungary

**Keywords:** chronic illness, dyadic coping, environmental self-regulation, home, qualitative study, grounded theory, Emotional Map of the Home Interview

## Abstract

Environmental and emotional self-regulation skills play a critical role in promoting well-being of individuals and in encouraging healthy relationships. However, occurrence of chronic illness in one family member complicates routine dyadic coping processes for the couple. Additionally, according to environmental psychologists, self-regulation processes are influenced by individuals’ perceptions of their socio-physical environments, and during times of chronic illness, the family home is frequently the primary site of dyadic coping. To date, few researchers have investigated the complex relationship among dyadic coping, the family home, and self-regulation processes in the context of chronic illness. The purpose of this paper is to report the results of qualitative research conducted to explore these relationships by analyzing participants’ emotionally significant experiences within the family home. We purposively sampled and conducted in depth semi-structured interviews with 23 adults representing 10 families with one chronically ill adult family member. Representative illnesses included epilepsy (4) and chronic back pain (6). We used the Emotional Map of the Home Interview method (EMHI), an elicitation process in which participants are initially asked to place predefined positive and negative experiences on drawn diagrams of their homes. We analyzed the data through grounded theory coding methods, including open, axial and selective coding. Results of data analysis suggest that the family home operated as a critical socio-physical environment and had a profound impact on environmental and emotional self-regulation as well as on dyadic coping when one partner experienced chronic illness. Key selective codes derived from the data that reflect the variation and nuance within this impact included: “stress communication through the home space,” “coping by spatial separation” and “coping by joint striving for at-homeness.” These results reveal formerly hidden aspects of dyadic coping with chronic illness: the role of environmental cues, represented by the family home in this study, in perceptions of stress; the coordinated use of spatial-environmental contexts to engage the appropriate self-regulatory strategies for coping with illness-related stress. These findings demonstrate the utility of EMHI as an assessment tool and provide meaningful theoretical and practical information about dyadic coping among couples living with chronic disease.

## Introduction

Environmental and emotional self-regulation skills play a critical role in promoting the well-being of individuals and in encouraging healthy relationships (Korpela, 1989). The occurrence of chronic illness in one family member not only presents additional challenge to environmental and emotional self-regulation for all family members, but also complicates routine dyadic coping processes for the couple. The purpose of this paper is to explore the dyadic coping processes in the family home in situations of chronic illness, and the impact of those processes on the couple’s relationships. To this end, we followed a constructivist grounded theory methodology (Charmaz, 2014) that allowed us to systematically approach data collection and analysis with the end goal of inspiring the development of a novel theory that is not based on *a priori* hypotheses and does not aim to confirm existing theoretical frameworks. Although Glaser and Strauss (1967) suggested that authors working to develop grounded theory avoid extensive review of prior literature, it is necessary and desirable to be able to define the constructs of interest, and to ensure that theory building efforts are unique and warranted. This research project was informed by the components of the Systemic Transactional Model of dyadic coping (STM; Bodenmann, 2005) in combination with environmental psychological accounts of coping with chronic illness.

### Dyadic Coping and Chronic Illness

Chronic illness is a life condition that in many instances also generates chronic stress. This makes constant coping efforts necessary, both for the individual and his or her social network. Appropriate coping with illness requires a series of coordinated behaviors that are either necessary for survival (e.g., medication adherence) or contribute to improvements in health and quality of life (e.g., regular exercise). Moreover, individual efforts are embedded in social bonds. Close relationships are especially affected by the illness; close others are involved in the coping process along with other aspects of illness management. Indeed, much prior research shows that health outcomes depend considerably on the availability and quality of social support and involvement of close relationships in the coping process (Martire and Helgeson, 2017).

The Systemic Transactional Model (Bodenmann, 2005; Falconier et al., 2016) of dyadic coping posits that relational coping with stress occurs in circular chains of perceptions and reactions of the partners to each other’s signs of stress and to the resulting actions. The most important elements of the process are stress appraisal and stress communication by one partner, the perception of this (verbal or non-verbal) communication by the other partner, and his or her corresponding coping reactions; by definition these coping efforts may be positive or negative. The cyclical nature of the process entails the perception of these reactions by the stressed partner, which again affects by relieving or amplifying the experienced stress. This circle may be continued until some type of resolution occurs. STM also includes joint efforts of the partners, referred to as *common dyadic coping*, that are engaged to handle common challenges and illustrate the systemic, mutually interdependent nature of the joint coping efforts in couples.

While STM was originally developed to model coping with daily stress and adversities in couples, it has been increasingly applied to chronic health conditions (Falconier et al., 2016). Prior research on dyadic aspects of coping with chronic illness has focused primarily on three interrelated but distinct themes. The first of these is maintenance of a high quality, functioning relationship in the context of a chronic and often life threatening stressor (i.e., a chronic illness experienced by one partner). Empirical studies have reinforced theoretical assumptions that more frequent partner use of positive and supportive, as opposed to disregarding and negative dyadic coping processes, is associated with better individual mental health (Meier et al., 2011; Regan et al., 2014; Vaske et al., 2015), and relationship functioning (Badr et al., 2010). In a systematic review of 33 articles on dyadic coping in the context of cancer in one partner where relationship functioning was an outcome, pooled results confirmed that open and constructive communication, in conjunction with positive dyadic coping of the partner, was associated with better relationship functioning (Traa et al., 2015). Alternately, dysfunctional communication (e.g., protective buffering, demand-withdraw communication), and patterns of negative dyadic coping of the partner (e.g., hostile behavior and blaming) were associated with lower relationship satisfaction.

The second prevalent theme in prior research is exploration of how dyadic coping strategies may affect both patient’s and partner’s adjustment to the illness itself, including aspects such as self-management of the illness, treatment related decisions and health behaviors. Previous research suggests that better dyadic coping of the partners was related to better health management of the illness by the patients (Helgeson et al., 2017). Interestingly, Helgeson et al. (2017) also studied implicit communal coping, measured as first-person plural pronoun usage during a diabetes discussion, and found that higher implicit communal coping of the partner predicted better self-management in the patient. They concluded that those communal coping efforts of the partners that, on face, appear less obvious might be especially beneficial for the patients.

In other research, the partner’s dyadic coping also predicted his or her adjustment, although results were inconsistent. In one study with partners of patients with breast cancer, hostile dyadic coping predicted men’s higher detachment and alienation from the illness experiences of the female partners, that is, they felt their partners’ illness to be more intrusive (Feldman and Broussard, 2006). In another study focused on CVD patients and their partners directly after a CVD event, Bertoni et al. (2015) found that greater involvement in illness self-management was associated with higher negative coping, e.g., withdrawal, of the partner. These mixed results indicate that coping and adjustment is a complex process (c.f., Berg and Upchurch, 2007) that might have unique challenges and possibilities in certain phases.

The third prevalent theme described in prior dyadic coping in chronic illness research includes studies focused on an underlying quality of the couple’s relationship that recent conceptualizations refer to as “We-disease.” The term refers to a common appraisal of the illness by both partners as well as inclusion of the illness in the joint concept of the relationship. This view of the illness by the partners as inherently shared responsibility may promote both coordinated efforts and emotional sharing; these are two relational qualities that were found supportive for well-being, recovery and health maintenance in couples (e.g., Coyne et al., 2001). Empirical data support this reasoning by showing that more shared representation of the illness in the relationship is associated with better cooperation, more constructive dyadic coping and better quality of life (Kayser et al., 2007; Berg et al., 2008; Helgeson et al., 2017).

### Chronic Illness in the Family Home

There has been a tendency in developed countries to strengthen the role of home care and to relocate several health services to family homes (Williams, 2002). Examples include provision of support for early mother–child relationship (Olds et al., 2002), especially in disadvantaged families (Fraser et al., 2000), home use of certain diagnostic tools (e.g., in sleep disturbances in children, Nixon and Brouillette, 2002), analgesic for chronic pain in the home (Beyer and Simmons, 2004), and home birth (Bailes and Jackson, 2000).

The home environment in chronic illness is conceptualized as the *caring landscape* (Williams, 2002) or *therapeutic landscape* (Dyck et al., 2005). The conception of therapeutic landscape refers back to the transactive nature of the relationship between a natural setting or landscape and the person, where meaning and health generating effects of the landscape are in a mutual transactive relationship with human agency. Williams suggested a holistic health paradigm that is equally concerned with physical, mental, emotional, spiritual, environmental and social factors and encompasses their interactions (c.f., Bell et al., 2018). Moreover, individuals are drawn to places that facilitate “restorative experiences” (Korpela et al., 2001, p. 573) following stress-inducing experiences. Therefore, an individual’s home as a sociophysical environment might play several roles in the coping processes with chronic illness and the self-regulation challenges during this process. The home also has potential to greatly influence health in positive or negative ways, depending on the tone of associated emotions and relationship experiences (Manzo, 2003, 2005; Dyck et al., 2005) and the nature of perceived changes in the meaning of home (Donovan and Williams, 2007).

Chronic illness challenges the patient’s exiting relationship with the home; this challenge extends to patients’ social networks. Research with chronically ill persons repeatedly found they engaged in processes such as restructuring of routines and meanings relating to the home (Dyck et al., 2005; Donovan and Williams, 2007). Simultaneously, individuals with chronic illness make efforts to maintain their pre-illness identity through active management of the home to enable private and social activities. This requires efforts to balance conflicting priorities, for example, maintaining the privacy of home while receiving homecare, or choosing alternately to display or to hide symbols of pre-illness identity and experiences (Mærsk et al., 2018).

Researchers have identified several distinct processes by which individuals cope with the challenge of navigating this changing role of the home that results from chronic illness. Corbin and Strauss (1985) described three types of work, i.e., psychological tasks related to the restructuring of home experiences, and their interplay. These include *illness work* to handle illness related challenges, *everyday life work* to handle the tasks of living a manageable life even in the face of adversity, and *biographical work* for recreating the life narrative. Another process is *orchestration*, meaning the management of several elements of home care by spousal carers concurrently and with considerable effort and precision (Karasaki et al., 2017). It is important to note that certain aspects of the experience with the home may become especially important and at the same time fragile and vulnerable: self-expression, control, security, and restoration (Downing, 2008).

Tamm (1999) and Öhlén et al. (2014) also assessed changes in the role of the home that result when chronic disease or illness is present. Öhlén et al. (2014) categorized individuals’ responses to illness at home into the following: “(i) being safe, (ii) being connected, and (iii) being centered.”^1^ Tamm was additionally concerned about changes that occur when home treatment accompanies chronic disease, and questioned whether illness at home might not lead to revisualization of the home as something that more closely resembles an institution. Importantly, studies also indicate that the process of restructuring is far from being linear; instead, it is inherently stressful and followed by struggles both at individual (Riley et al., 2001) and relational levels (Downing, 2008; Moore et al., 2013; Årestedt et al., 2016; Karasaki et al., 2017). This stress almost inevitably involves coresidential relatives (Corbin and Strauss, 1985; Donovan and Williams, 2007; Årestedt et al., 2016), although influence of these individuals may be disproportionately more burdensome for woman caregivers (Allen and Webster, 2001; Piercy, 2007).

In summary, prior research suggests that individual and relational aspects of coping with chronic illness unfold in transaction with the family home as a complex sociophysical environment. Dyadic coping processes as described in the STM (i.e., stress appraisal and stress communication, coping responses and their perception of the partners, and common dyadic coping efforts) are specific and important types of relational coping with chronic illness. Therefore, we may assume that dyadic coping in the family home implicitly or explicitly involves not only the partners but also broader socio-physical environmental aspects. However, while the findings presented above provide helpful theoretical insight, we were unable to identify any prior published examples of research reports in which authors attempted to explain the precise interrelation of chronic illness, dyadic coping and environmental self-regulation processes in the home. This gap in the existing literature supports our exploratory and theory building approach.

### The Present Study

To explore the dyadic coping processes that unfold in the context of the homes of couples living with chronic illness, we applied a constructivist-interpretative research paradigm (Lincoln and Guba, 2000; Ponterotto, 2005, 2010; Ylikoski, 2013) and the grounded theory methodology (Strauss and Corbin, 1998; Charmaz, 2014). The former assumes that interactions and constructions in a social world result in multiple realities that are equally valid, while the latter encourages intensive dialog and intersubjectivity between researcher and interviewees to explore meanings and experiences. A qualitative research strategy that fits into the constructivist-interpretive methodological paradigm has a “contextual” character that makes it especially efficient for exploring complex social phenomena. Specifically, contextual research does not rely on previously selected and defined variables; instead, research encompasses any variable that emerges through the research process.

The research described in this report reflects a focused re-analysis of data originally gathered for the first author’s Ph.D. thesis (Sallay, 2014), which generally explored emotional self-regulation among family members when one partner experienced chronic illness. Analyses of these data began to reveal the critical role of dyadic coping in the home environment, which led to development of our research question for this current study: *How do families with a chronically ill member use dyadic coping processes in the context of the home environment?*

## Materials and Methods

The present research was designed to elicit detailed information from interviews facilitated by a visual elicitation method. Participants included adult patients with chronic illness (epilepsy and back pain) and their adult family members living in the same home. We used the “Emotional Map of the Home Interview” protocol (EMHI; Sallay, Martos, Chatfield, Dúll, in preparation), a drawing procedure followed by a semi-structured in-depth individual interview. The governing institutional review board granted approval for the procedure.

### Sample

In order to find answers to the research question and subsequent questions derived from the coding process we purposively sampled 10 persons who had epilepsy or chronic back pain of any type, for at least 1 year, and who resided with at least one adult family member. The final sample consisted of 23 Hungarian adults from 10 families: four families with a person affected by epilepsy, six families with a person affected by chronic back pain. The sample included 13 women and 10 men, aged between 25 and 57, with two families residing in a rural area and the other eight living in a large urban area. See Table 1 for additional demographic details.

**Table 1:** Characteristics of the sample.

ID	Family member	Age in years	Education	Residency	Y’s living there	Description of family
1E-1	Mother	27	Other	Budapest	18 years	Three generations, single mother, one child
1E-2	Grandma	53	University		18 years	
1E-3	Older brother	29	College		18 years	
1E-4	Younger sister	25	Other		18 years	
1E-5	Grandpa	53	University		18 years	
1G-1	Wife	41	n.a.	Budapest	9 months	m. couple
1G-2	Husband	43	University	Budapest	9 months	
2E-1	Wife	27	College	City in country	9 months	m. couple with one child
2E-2	Husband	31	College		9 months	
2G-1	Wife	36	College	Bp. surr.	4 years	m. couple with one child
2G-2	Husband	37	College		4 years	
3E-1	Husband	28	University	Budapest	3 years	Cohabiting couple
3E-2	Wife	25	University		3 months	
3G-1	Wife	35	University	Budapest	6 months	m. couple with two child
3G-2	Husband	34	University		6 months	
4E-1	Daughter	29	High school	Budapest	3,5 years	Mother and adult daughter
4E-2	Mother	57	College		4 years	
4G-1	Wife	40	College	Bp. surr.	7.5 years	m. couple with two child
4G-2	Husband	43	University		7.5 years	
5G-1	Wife	29	Skilled worker	Budapest	5 years	m. couple
5G-2	Husband	32	Skilled worker		5 years	
6G-1	Husband	36	Other	Budapest	8 years	m. couple with one child
6G-2	Wife	37	University		2 years	


*The index patient in the family has the number 1 as last digit (e.g., 3G-1); type of illness is coded as E (epilepsy), or G (chronic back pain); numbering of the family: first digit. fam.member, family member. In Hungarian higher education, college refers to dedicated technical or vocational training, while university education is more focused on sciences and humanities. Bp. surr., Budapest surroundings; m.couple, married couple; n.a., no answer.*

### Data Collection

**FIGURE 1:**
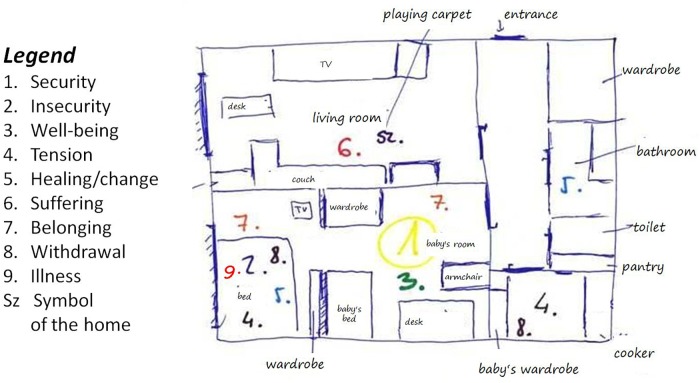
The Emotional Map of the Home: an example.

The interview guideline followed the EMHI protocol. Sallay (2014) developed the EMHI with the aim of exploring emotional and environmental self-regulation processes in the space of the home. The interview begins with the “anamnesis of the homes,” that is, a process of guided recall of previous homes of the person along with the associated emotions. As a second step, we asked subjects to draw a layout of the home indicating functions and important furniture within each room. We included nine emotionally important self-regulation experiences in the interview guideline: (1) security (2) insecurity (3) well-being (4) tension (5) healing/change (6) suffering (7) belonging (8) withdrawal (9) illness, and we asked participants to mark the place(es) of these nine emotionally important self-regulation experiences on the layout (e.g., “Where is the place of security for you in your home?”). We also requested participants to add a tenth item that reflected an encompassing symbol of the home (see Figure 1 for an example of the layout with the places of emotionally important experiences marked on it).

We asked for stories relating to every place they marked on the map (e.g., “What stories are associated to the way you experience security in the kitchen?”). The length of the interviews varied between 43 and 82 min. The first author completed all the interviews. Eighteen participants were interviewed in their homes, three participants were interviewed in the researcher’s university office and two participants completed interviews in their respective workplaces. All interviews were tape recorded and transcribed verbatim.

Although this research was informed by constructivist grounded theory design (Charmaz, 2014), for practical and resource considerations, we did not fully employ all strategies associated with classic grounded theory methods. We focused on purposive sampling, prioritizing selection of participants who reflected the experience and situations of interest. Throughout the interviews we used the EMHI protocol and we did not extend data collection to the point where additional data analysis fails to yield new information (i.e., saturation). Nevertheless, we incorporated processes of theoretical sampling in our analysis, including reflexivity and iterative comparison between new and existing data (Charmaz, 2014).

### Data Analysis

We followed the principles of grounded theory methodology to analyze the interview transcripts (Strauss and Corbin, 1998; Charmaz, 2014). A hallmark of grounded theory is use of theoretical sampling, which means we concurrently and iteratively engaged in processes of data analysis, comparison of new with existing data, and reflective consideration and re-consideration (Charmaz, 2014). As a result of these processes, concepts that emerged or were identified in analysis directed subsequent assessment as well. We applied a hierarchical, inductive coding process (“open coding”) to each meaning unit, and developed analysis toward increased abstraction. In keeping with grounded theory tradition, we considered each line of the transcript in search for meaning units, such as sentences or paragraphs that included information referring to our research question. During the coding phase, we searched for connections, similarities and differences between meanings by means of an explicit constant comparative method (Glaser and Strauss, 1967). Raising and organizing of thematic units was made in a three level hierarchical coding process (open, axial and selective codes; Strauss and Corbin, 1998; Hallberg, 2006; Charmaz, 2014). While progressing from initial open coding to axial coding and then to selective coding, we continued to engage in theoretical sampling processes. For this analysis, theoretical sampling centered on revisiting and reflecting on data and codes, to refine axial and selective codes when categories appeared unclear or incomplete. In grounded theory, research notes are referred to as *memos*. We used memos in each stage of interaction with data, including the levels of coding, to record and crystallize the processes. We referred back to these memos later in the process to help describe and refine the emerging theoretical concepts. Our coding process eventually resulted in a three level “code tree” that contains the selective, axial and open codes (see Appendix).

## Results

Following, we provide the code tree with the selective and axial codes (see Table 2) and an expanded description of several subparts of the three primary selective codes. We validate these codes with supporting excerpts from participant interviews (translated from Hungarian).

**Table 2:** Code tree.

Code no.	Selective and axial codes
1.	Stress communication through the home space
1.1.	Direct stress communication is lacking – stress appears indirectly through space use
1.2.	Open disagreement focuses on use of space
1.3.	Differences in partners’ priorities for space use cause conflicts
2.	Coping by spatial separation
2.1.	Conscious distancing in coping with the symptoms
2.2.	Distancing in the coping with relationship stress
2.3.	Mutually reinforcing processes of distancing
3.	Coping by joint striving for at-homeness
3.1.	One partner takes care of the other in the absence of stress communication
3.2.	The risk of taking over home duties
3.3.	The common use of the home space supports coping with stress
3.4.	The joint shaping of the home space


Our analysis revealed that everyday dyadic coping processes were embedded in partners’ routine movements within the home space and in the ways they used the home environment. These movements and interactions within the home environment played a prominent role in dyadic interactions, stress communication, and couples’ attempts to cope with stress stemming from the symptoms of the chronic illness as well as from other areas of their relationship. In this grounded theory analysis, we elaborated three main categories, or selective codes, which describe (1) processes of stress communication through the home space, (2) dyadic coping by spatial separation, and (3) dyadic coping by joint striving for at-homeness. Just as ways of stress communication are characterized by specific person–environment interaction patterns, the dyadic coping processes also involve more or less conscious, and more or less ritual ways of creating spatial separation or, inversely, patterns of seeking closeness to each other in the space of the home.

### Stress Communication Through the Home Space

One component of the process of dyadic coping at home is demonstrated by couples’ behavioral patterns through which they involve the environment in communicating or in hiding their stressful feelings. In these experiences, stress communication either extended beyond verbal messages and was manifested by movements in space or it became apparent in the partners’ discourse about the home space. The application of the EMHI protocol elicited explicit descriptions of stress communication through movements in space. Partners routinely used physical distance and specific places to express their stressful feelings. In addition, physical environment gained also symbolic significance when couples expressed relationship stress through negotiating the rules related the use of the home environment. Three variations of these phenomena emerged: lack of direct stress communication, with stress instead indirectly communicated through use of space; open disagreement focuses on use of space; difference in partners’ needs for space use causing conflicts.

#### Direct Stress Communication Is Lacking – Stress Appears Indirectly Through Space Use (Axial Code 1.1)

In order to preserve the harmony of family life and to save children from witnessing parents’ conflicts, some couples expressed the shared belief that tension cannot appear during the time spent together. In this case, partners had to find ways for dealing with their stressful feelings outside of the other partner’s presence in the common home. For example, one wife (a young mother) described how she regularly relieved the tension she felt by praying alone in the living room after her husband had fallen asleep in one of the bedrooms:

For me, the tension is connected to not sleeping. These two are related, so during the day I can mostly hold back the inner tension, usually it comes out when I either have to sleep or have the possibility to rest. So for me it’s pretty much connected to the bedroom and sleeping, because during the day I can mostly hold it back somehow. So it’s mostly in the evening when…I don’t even have the time, by the way, to deal with this [tension] during the day.

This couple reported spending most evenings separately while dealing with daily stress. Another variation of missing stress communication occurred when chronically ill wives and husbands asserted that there was no suffering in chronic illness and their condition was not an illness but a specific “state.” In their case, there was no need to take distance from each other; they managed to hide stress in the presence of their partner.

In contrast, however, some wives expressed the need to communicate their stressful feelings to their partner but believed the partner could not respond to this need, so these conversations were ineffective. A young mother described how she regularly failed to discuss the problems with their flat being too little for the family:

I usually only talk to my mom about these things. And I’m trying with daddy [her husband] too, but, but… sometimes it only makes us fight, if something like this… no… I stopped now a little bit to talk about this moving thing, but… Actually the most important would be that I’m with him. So that’s the most important for me… So this idea of where to live, this shouldn’t matter, but unfortunately it actually does matter.

Consequently, their shared bedroom became a place of tension for the husband: “Every night when mommy wants to talk, but daddy wants to sleep, and that’s where the problems begin at the end of the day.” Couples, who experienced difficulties in discussing stressful topics overtly, also described the behavioral pattern of taking turns when using home spaces. For example, some reported preferring to cook alone in the kitchen or staying in the living room when their partner was not there.

#### Open Disagreement Appears in Use of Space (Axial Code 1.2)

Several couples experienced that stress communication was regularly expressed in the form of disagreement. Couples, who reported having frequent disagreements on stressful issues, also described the role of the home space in these exchanges. One variation of this was that the partners were regularly using certain spaces of their home in opposite ways, and this frequently led to quarrels. In the following excerpt, a young husband explains how he was fighting with his wife to gain control over the use of the home space:

Well, there is this tension [regarding the closet] because mum [his wife] wishes to keep everything and I want to throw out everything we do not need. It usually leads to problems, but… we can manage them… I decide what I would throw out and therefore… (laughter), and that is it.

In some cases, couples explained that direct quarrels were ritually ended by one partner withdrawing to a distant or separate part of their home. One couple, for example, reported they spent most of their shared time in the living room, except for those regular moments when they entered into a disagreement and the wife withdrew to the bedroom alone. This way the bedroom often became primarily a refuge for the wife, and not a shared retreat.

Disagreements and quarrels also played an important role in the evolution of additional aspects of the relationship. This couple, who reported having regular disagreements while in bed together, also described the failure of their sexual relationship.

#### Differences in Partners’ Priorities for Space Use Cause Conflicts (Axial Code 1.3)

Under certain circumstances the home environment itself could became a source of chronic stress for couples. For example a couple with young children had to make a consensus decision to choose a new flat, but the partners expressed different needs. Eventually one partner conceded her preferences in the final decision. But after settling in the chosen flat, the unbalanced decision continued to play a role in their everyday conflicts.

How space within the home was used could also lead to emerging accusations that caused long lasting areas of instability in the relationship. The following excerpt illustrates circularity in the flow of dissatisfaction related to space use. While drawing the layout of her home, one wife expressed her dissatisfaction with the physical environment (i.e., there was no space for her belongings after moving in) that also conveyed her dissatisfaction with the relationship as a result of feeling underappreciated:

And this is the living room. Well, these sizes, they really seem rather disproportionate but, well, here is a small wardrobe. Does it matter that my things do not fit? I could provide an excellent drawing of my things that do not fit anywhere. There are certain items that have been in the same box since I moved in many years ago. Therefore, it causes a little…Generally arguments. (…) And there is also a set of wardrobes which, of course, is not mine [it is my husband’s], hence I will draw it small. Yes. (…) I have to say it is a bachelor’s flat. It was designed as such and has changed a little since I arrived, and I have not been able to make any radical [changes].

Another wife described how control fight for the possession territory led to a ritual of repeated conflicts in their relationship. This wife had a computer desk in one corner of their living room where she could work and the husband had a working room on his own. Still, the husband had the habit of using his wife’s computer instead of his own:

But still, the end of this was him always being here [at the wife’s working corner]. And I couldn’t get to my computer just to check an e-mail or look up for something online, so for me this is like… the feeling was that it compromises my liberty, that I can’t do it anytime, while we made the whole thing so that there wouldn’t be any problem… And then I got upset, and then he said to me not to be angry, and he would move over, and then, after this, there was something again, which is why he slowly came back to my place. And then now too, for a lot of times, I don’t know why, but in the last few days he’s there, in his own place. Yes. And he would come over here just a few times, which is okay. But like… the books were in heap like this and I couldn’t even be there comfortably, so that I’m like claustrophobic too and it was tightened, the possibility to get in between the books. So for me this is the tension, when I saw that he sat down there again, I could feel the anger in me.

As with the prior example, these partners assume asymmetric roles in the debates: the husband clings to his ways of using the home space without addressing his wife, while the wife eventually respond by verbally expressing her stressful feelings resulting from this.

### Coping by Spatial Separation

Coping with stress in the space of the home was strongly linked to variations of couples’ spatial separation throughout the interviews. For some couples, spatial separation appeared to result from one or both partners’ conscious decisions, and was a strategy used to cope with the ill person’s symptoms. In other instances, spatial separation seemed to occur when partners experienced tension in the relationship, and resulted either from a conscious decision or from the couples’ everyday movements. In this latter case, separation was not necessarily the explicit goal expressed by the couple, but still it occurred ritually and played an important role in preserving emotional stability in the relationship. In the third pattern of spatial separation, the need for separation stemming from a tense relationship was intertwined with the need for separation resulting from the illness experience.

#### Conscious Distancing in Coping With the Symptoms (Axial Code 2.1)

Couples elaborated several ways of finding distance from each other in the home space in order to deal with the ill partner’s symptoms. Our analysis revealed that the phenomenon of conscious distancing occurs with varying intensity. In some cases it consisted of the ill partner regularly but briefly retiring alone in one room to alleviate his or her symptoms (for example the wife with chronic back pain taking hot shower while her husband is having dinner with their child). In another case, parents of a young adult living with epilepsy held the belief that paying extra attention to their daughter’s symptoms is the wife’s duty, so she remained close to their daughter; the mother and daughter slept in the same bedroom and the husband used the other bedroom.

Another wife with chronic back pain explained how sleeping separately from her husband helped her alleviate the symptoms: after sleeping in the shared bedroom:

I limp here down the stairs every morning. Then it [the pain] goes away somehow, when the day begins, but when I get out of bed and I have to go out, that’s basically… that’s like the death itself until I can walk down the stairs. And it’s very interesting too, that we don’t live together as husband and wife, we function as father and mother. We have this common agreement and actually I don’t sleep here in the night (points to the bedroom), I sleep here in the guestroom with my little son and ever since we began to sleep here, the pain’s gone.

In their case, both partners expressed their conscious consent to use separate bedrooms in order to ameliorate the wife’s symptoms.

Separation was yet more intense for those couples when husbands stated they had “nothing to do” with coping with their wives’ illnesses. In one case, the husband explained that he could not have any impact on the evolution of the wife’s chronic back pain. Another husband held the belief that the wife’s chronic back pain was rather a “projection of her inner state” than an illness, adding “everything starts in your head.” In both cases, according to husbands’ perceptions, the wife had the responsibility to manage her illness alone, at any moment during the day and at any given place in their common home.

#### Distancing in the Coping With Relationship Stress (Axial Code 2.2)

Coping with relationship conflicts often implied the couple ritually taking a certain distance from each other in the home space. Spatial distancing occurred on different scales from withdrawal inside the shared bedroom to creating an alternative home in another quarter of the city for one of the partners.

One couple gave the name “pouting bed” to a piece of furniture in their bedroom, as an example of a ritual of taking distance:

When we had a row, one of us moved to the pouting bed. It has begun with my wife, and then, she slept there twice, and then I also tried it once. But after an hour or so, and after that, we spoke again. We had the feeling like wanting to go back after an hour, but our pride set us back.

In case of one couple, who were parents of a girl of kindergarten age, the ritual of taking distance appeared to separate them throughout their entire house. This behavior pattern required a high degree of co-ordination between them. They had two bathrooms and two TVs in their two-story family home that they divided between each other; they additionally avoided being together in their bedroom in the evenings while the wife was awake. This is how the wife (living with a chronic back pain) explained it:

To me withdrawal is when my husband puts our child to bed and sometimes I am asleep half an hour later. But I also enjoy going to bed with my laptop to quickly browse the news in approximately 1 h. I do that when I am on my own. Because I don’t always want to watch the same TV program as my husband. In such cases, I am upstairs, he is downstairs, which is slightly bad because this way it leads to, how should I put it, separation. He prefers Spectrum and National, i.e., educational programs, and I may be a complete idiot, but I watch Dallas even these days, and that drives my husband crazy, and asks me if I am out of my mind because I watch Dallas. I keep saying that I watch it only as background entertainment, because I do something else at the same time but I like having approximately 1 h like this after the great noise has calmed down.

Their alternating use of the home space, which also extended to the bedroom for a part of the night, had evolved partly as the consequence of the husband helping his wife look after their child and do the housework (because of her chronic back pain), and partly as a way to avoid repeatedly facing the problems of intimacy between them. The wife’s explanation also reflects some concern about the growing distance between them (“it is slightly bad because this way it leads to, how should I put it, separation”). Their separate ways, driven by their choice of TV programs, seem both reflecting their individual needs and their common way of coping with the tension in their relationship. However, this coordinated pattern of using the home space also imposed some threat according to the wife’s explanation and was accompanied by the failure of their sexual life as reported by the husband.

Patterns of separation in couples’ coping with relationship stress also appeared to be linked to childhood experiences. One young mother used the spatial metaphor of being in a “castle” while speaking of her feelings of security experienced in her baby’s room:

I feel best here, in this room. Here can I find myself and everything I wanted the best. In this small, approximately 3×3 [meters] room. Even as a child, I always liked building a small castle and hiding there. This 3×3 room was more or less the same as a small castle.

In her fantasy, their baby’s room turned into a sound fortress, which would both protect and separate her from her husband whom she resented. This way, her perception of the situation was guided by an image of her home as a child. This perception, in combination with other experiences became the basis of withdrawal from the relationship by retiring into her baby’s room.

In the presence of escalating conflicts and relationship crisis, one couple’s need for coping by spatial separation was so strong that they decided to rent a separate flat for the wife where she could retreat either with one of their children or alone. The wife explained that she always felt relaxed and secure there and elaborated a special attachment to this place – she called it her “snug” and described how she greeted it every time she arrived there.

#### Mutually Reinforcing Processes of Distancing (Axial Code 2.3)

Patterns of separation in coping with the symptoms of a chronic illness appeared to be inseparably intertwined with patterns of coping with relationship stress. For example, a couple living with the wife’s chronic back pain described experiencing a growing distance between them in the course of the construction of their family house. Both partners described their respective situation as being abandoned by the other, and each felt they had to assume all responsibility for the works and had been required to engage in physically demanding duties. Their experiences were linked to the wife’s symptoms in a way that restrictions of the wife’s capacities became a basis for mutual blaming and consequently resulted in a growing need for distance between them.

Coping with the symptoms of the illness also implies having special environmental needs, for example a place for morning exercise or a hard mattress for sleeping. One husband’s need for a hard mattress became a source of conflict in the intimate relationship of a young couple living with the husband’s chronic back pain. In describing their problems with sexual intimacy, the wife explained:

But in my opinion this [sexual problems] is a thing which causes tension. And I must add that this bed, it’s a very hard bed and I never agree on this with my husband. He thinks that hard bed is the good bed, but I think that the not too soft, but still softer than the one he sleeps on, because that’s a hard-as-concrete bed, I have to say, I should use this expression. And on that side of the bed where I sleep I have an additional thin sponge too. So I couldn’t sleep so much, I wasn’t able from the beginning, that… we couldn’t agree on this either, that what the bed should be like.

In this instance, the home environment intended to support the husband’s recovery created a chronic conflict and a physical distance between partners.

### Coping by Joint Striving for At-Homeness

The third component of dyadic coping process in the interviews was patterns of coping by coordinated actions aiming at creating feelings of relational and physical security accompanied by emotional and physical care – what can be called the sense of *at-homeness* (c.f., Seamon, 2000). Some of the partners’ common actions proved to be demanding or burdensome, such as one partner taking care of the other even in the absence of stress communication, or taking over additional duties at home. Other actions of common coping, such as the common use of the home space and joint shaping of the home were associated with more positive feelings like joy, amusement, relaxation and pride.

#### One Partner Takes Care of the Other in the Absence of Stress Communication (Axial Code 3.1)

Several healthy partners tended to care for the other even in cases where the ill partners were hiding the stress resulting from their chronic symptoms. In case of a young couple living with the husband’s epilepsy, the wife explained that she only felt safe when they were at home, because it was in the home space where she could care for her husband in the moments of his seizures. In contrast, however, in the husband’s perception, their home did not have any special significance for his feelings of safety. Another husband, speaking of his wife’s seizures, demonstrated greater feelings of stress and concern than his wife who was the one actually experiencing the seizures. This husband was especially worried about his wife’s loss of control whereas the wife was more confident in her ability to manage the situations.

In addition to feelings of worry, one husband initiated therapeutic and healing activities for his wife who had chronic back pain but use a coping pattern based on hiding her pain. The wife explained:

For me it was an absolutely normal part of my life, I didn’t even bother, it hurts, it has always been hard and now it’s getting even harder and it’s like part of the life, and well what, others have pain in their leg. And then he [my husband] started this, that it doesn’t have to be like this, I don’t have to accept this, I should start to go after this, and I should find, so I should see an expert and I should care about it. So like, he pushed it that I have to take a step forward.

The wife accepted the suggestion to see a doctor and the husband went on to initiate the home ritual of the morning therapeutic exercise, for which they get up earlier together. The husband was experiencing the positive outcomes of their common coping:

She is used so much to living with this thing that she doesn’t say a word normally about her having a problem. This made such a difference, that we wake up earlier in the morning because of her training… I don’t like to wake up early but I do appreciate it a lot that she wakes up.

#### The Risk of Taking Over Home Duties (Axial Code 3.2)

While most healthy partners tended to take over household duties from the ill partners, paradoxically these actions also involved certain risks for relational or individual well-being. In some cases, the ill partners considered the quantity of duties taken over insufficient or in other cases found the partner’s help inefficient. In either instance, partners reported that blaming followed the healthy partner’s actions. This is how a wife living with chronic back pain described an everyday scene when she arrives home and finds dirty vessel in the sink: “Well I let the tension out, and then [the husband says], ‘it’s okay sweetheart, I’m coming,’ and then really, so it’s solved, so no, there isn’t any conflict because of this… Okay, I could stop myself from doing the dishes but sometimes not, so I start it and then he says ‘but sweetheart, it’s my turn’ and, well I’m telling him ‘you were at home, anyway.’ Whatever, forget it.” In this case, the offered help from the husband’s side and a low-key blaming from the wife’s side are present at the same time. The wife appreciates the way of help but is dissatisfied with the timing of the offered help.

The presence of chronic illness makes it necessary that healthy partners increasingly take over household duties for longer periods of time. This means of dyadic coping can become especially burdensome and exhausting with time. A husband of a young mother with chronic back pain explained how he struggled with this condition:

For me it’s not, I don’t say that it isn’t hard, it is hard but… I won’t become a martyr just because I have to vacuum or because I have to carry upstairs the washed clothes so she can hang them out or I have to wipe up the dust or lift up something and put it into the cabinet or get down something or just lift something. So this isn’t hard, it’s normal… However in these last times my back hurts too. I don’t really understand it. First the crackle and then now the pain starts to come out. But I don’t think so that I’m going to be sick.

While steadily taking over duties from his wife, he seemed to be uncertain whether his feelings of being overloaded could be appreciated without having to assume the role of the “martyr.”

#### The Common Use of the Home Space Supports Coping With Stress (Axial Code 3.3)

In contrast to couples who described patterns of coordinated avoidance and separation, others emphasized the positive effects of being close to each other, moving together in the space of the home either in presence of the children or, in the case of childless couples, the couple themselves spending their time together at home. For a young man living with epilepsy, the sense of healing was everywhere – in their flat and on the terrace – due to being close to one another during the time spent at home:

On the terrace we don’t always need wine for this [healing]. The last time we just sat down and laid down and we were watching the sun and the city and we were just talking. Maybe we drank some water and cola, but that’s it. And we were just joking around and we do everything on the terrace, last time I sprinkled her with water from the hose so (*laughing*) so it’s like everything, really, we laugh from our heart.

The importance of their physical closeness is also reflected in the woman’s description:

We’re together in the bathroom as well, so basically always…So for example when one of us has something to do and the other doesn’t then… like then we use the workroom/den, but except that, really, we’re almost always together because if we aren’t, if for example one of us is cooking and the other is in the living room, even then we’re in the same space, so like a lot…

This experience of closeness, provided by the home space, also assured security for the wife who was regularly worried about her partner’s seizures and the fatigue that follows the seizures.

#### The Joint Shaping of the Home Space (Axial Code 3.4)

Common dyadic coping activities can focus directly on shaping the home environment. Active and shared shaping of the environment not only brought positive feelings in the time spent together but also had a longer effect on the relationship as the couple had created a new environment that later on reflected their creativity and harmony. In some cases, even minor modifications could represent important changes in the environment. For example, a wife (with chronic back pain) in a childless couple reported that lately, she had opened the door of their storage room that was intended to be their child’s room after they gave birth. She opened the room’s door and invited her husband to eat some fruit in that room. Later she asserted that the deliberate alternative use of this space helped them with the process of conceiving a child.

The same couple described their symbols of their home very similarly: both partners expressed the feeling that the big wardrobe in their bedroom that was their common work that could be the symbol of both their home and their relationship. This is how the wife explained it:

Somehow this wardrobe too is a symbol of the flat renovation, how it went, that we decided everything together and how we were planning and how it came out… It was like we did it together, the creative stuff; and this wardrobe was made like this.

The husband also pointed out how the wardrobe was created to support coping with the wife’s symptoms:

I think that this wardrobe symbolizes pretty well how we related to this whole issue of the flat. So that for my wife it was an important thing that we could have big, well packable wardrobe, she put really big effort in the planning of the inside that what would be in which height, what would be easily accessible, how it should be divided. And I had some ideas for the appearance. So I think that it symbolizes our cooperation and this… the harmony of this common activity in our home. It’s also designed to fit to her waist pain, of course, so that the important functions wouldn’t be too low, as I said, because bending is hard…

This process of dyadic coping with the stress of the illness and the stress stemming from furnishing a new flat led to a creative work, which reflected their cooperation every time they entered their bedroom. Thus, a glance at this wardrobe had a protective effect on their individual and relational well-being ever after its construction.

## Discussion

In this study, we assessed the relationships among the family home, self-regulation processes and, more closely, dyadic coping in the context of chronic illness. Reflecting back to our research question: *How do families with a chronically ill member use dyadic coping processes in the context of the home environment?* It was our initial assumption that the family home as socio-physical environment contributed significantly to self-regulation and relationship regulation processes. Indeed, one participant’s described practice of greeting her “snug,” a separate flat obtained primarily for her use, illustrates one of our essential findings: homes, and/or spaces within homes can be seen as actors in environmental self-regulation processes, thus they play significant and profound roles in dyadic coping in the context of chronic illness. In interpreting our findings, we focus on how families with a chronically ill member use the home as part of their dyadic coping processes.

### Summary of Main Themes

The results of our data analysis suggested that participants’ experiences could be categorized into three broad areas: processes of stress communication through the home space, dyadic coping by spatial separation, and dyadic coping by joint striving for at-homeness. These main themes and the respective subthemes were interrelated in a way that the system of these codes and their relationships described the processes through which partners communicated directly or indirectly the stress they experienced, and demonstrated variations of dyadic coping by spatial separation or by joint actions in the home space. While in some instances partners’ dyadic coping by separation led to a relief from stress and pain (e.g., the wife who had no pain when sleeping apart from her husband), in other instances this pattern of coping led to an opposite outcome – escalating stress in one of the partners or both partners with consequent explicit or implicit stress communication. In a similar way, partners’ dyadic coping by joint strivings resulted in stress relief in the experiences of certain couples, whereas others experienced escalating stress as a consequence of joint actions (e.g., coordinated taking over of home duties resulted in the experience of backache in the formerly healthy partner together with the failure of their sexual relationship).

The partners’ perception of the outcome of their dyadic coping actions depended also on the temporal perspective: coping by spatial separation appeared to assure momentary stress relief for the wife who held the image of a castle for her baby’s room but she also described her growing dissatisfaction and tension in their couple relationship partly linked to the lack of sexual intimacy between them. In addition, some couples applied a combination of dyadic coping patterns of joint actions and coordinated spatial separation related to specific places inside the home: they cooperated well in the kitchen and the living room where the husband took over household duties from his wife, but they consistently avoided each other in the bathroom and the bedroom.

### Spatial Aspects of Coping in STM Perspective

While our approach and research question was informed by basic tenets of previous dyadic coping research, most prominently by Systemic Transactional Model of dyadic coping (Bodenmann, 2005; Falconier et al., 2016), we still took an interpretative constructivist stance toward qualitative data without any preformulated hypotheses. Therefore, our results are not mere demonstrations of previously described constructs but can be regarded also as potential extensions and reinterpretations of the original concepts. For example, it might be appealing to identify the three main themes (selective codes) of ‘communication through the home space,’ ‘dyadic coping by spatial separation,’ and ‘dyadic coping by joint striving for at-homeness’ with the three main aspects of the dyadic coping process within STM: ‘stress communication,’ ‘negative’ and ‘positive (including common) dyadic coping.’ Following, we consider these overlaps as well as the dissimilarities between our interpreted findings and the STM.

Based on our analysis, we suggest that the family home mediates dyadic coping activities by acting at times as a proxy of or filter for direct and explicit communication. The degree of mediation varies in association with partners’ responses to one partner’s chronic illness; the congruence between partners when approaching coping with illness additionally impacts use of home space. Congruence of responses occurred on a continuum. Located at one end is complete separation of response to chronic illness, as the spouses who asserted that management of illness was entirely up to the partner with the illness. A midpoint of this continuum includes empathetic responses, such as the spouse who facilitated the ill spouse in his or her disease management and self-regulatory processes. The other extreme end comprises collaborative responses, often included in the selective code *coping by joint striving for at homeness*, and reminiscent of the “We-disease” theme presented in the introduction of this paper (c.f., Kayser et al., 2007; Berg et al., 2008; Helgeson et al., 2017). One example of this is the couple who described co-designing their joint wardrobe.

#### Space Use and Dyadic Coping

Dyadic coping responses were frequently communicated through separate or shared use of space but are not necessarily constant; couples might concurrently occupy the same space, such as when sharing a meal, and adhere to a regular practice of separation in other activities, such as media use. Frequently, couples described engaging in planning or taking advantage of naturally occurring opportunities to be separate from each other. It is of interest that separation occurred related to various activities, but was described multiple times related to sleeping arrangements that at times also interfered with partners’ sexual relationships. One couple described their use of the alternative “pouting bed” when disagreements occurred in bed; this is a particularly clear example of use of home space as a proxy for verbal communication related to nighttime intimacy. These patterns of either negatively or positively perceived individual and common acts of dyadic coping can be seen as specific manifestations of stress communication processes as described within STM. Nonetheless, our approach informed by the tenets of environmental psychology revealed non-verbal, implicit ways of stress communication by space use. Apparently, the role and significance of this kind of spatial communication – along with other types of non-verbal communication – have been seldom the explicit focus of STM-based dyadic coping research, although it is inherently present in the foundations of the model (c.f., Bodenmann, 2005). Thus, these aspects deserve further investigation and may contribute to potential extension of the original model.

#### Control of Space

Multiple participants described dissatisfaction over lack of control of space (c.f., Barnes, 2006), either due to storage of excessive or unwanted items, or due to a partner using the other partner’s space, rather than his or her designated space, or due to perceived unequal allocation of space. In one instance, a participant who was unhappy with having less space for her personal possessions than her partner did, drew an explicit parallel between this perceived lower priority placed on her space needs with less appreciation of her in general. For two participants, including the spouse who procured her own “snug,” and another mother who described her baby’s room as analogous to her childhood imagined “castle,” there was security in having a distinct space under one’s own control. These findings echo but expand upon previous findings, which posited that dyadic coping varied through the lifespan (Berg and Upchurch, 2007), and was vulnerable to contextual factors (Bodenmann et al., 2015). Specifically, we found that available space, and perceptions of control related to available space, comprised explicit factors that might appear to be contextual but played a more significant role by being used as means of communication of coping responses.

Obviously, control aspects of space use are interwoven with the social-ecological context of actual relationships as well. Financial power of a family may significantly influence the potential availability of private spaces and equipments. This way, financial problems and limitations as a context may be partly represented in spatial-territorial stress in everyday relational behaviors. Moreover, we need to consider that ownership of a house or flat plays a key role in the financial strategies of Hungarian families (Toussaint et al., 2012) and families often take extra burdens to achieve this long-term goal. Experiences of the respondents, for example that of the woman who struggled for control in her *husband’s flat*, stem from this social-economical background where upward residential mobility is difficult for lower middle class parents. Personal and relational stress and coping behaviors in the family home – as reflected in the interviews and the codes – cannot be exclusively tied to intrapersonal and interpersonal processes but broader social-ecological contexts have to be considered as well.

#### Control of Closeness and Distance

The selective codes ‘spatial separation’ and ‘joint striving for at-homeness’ as spatially embedded forms of dyadic coping represent two characteristically distinct ways of responding to the challenges of chronic illness and the resulting relationship tensions. Strategies under the code ‘spatial separation’ often involve actions of distancing, withdrawal and even lack of sexual encounters while ‘joint striving for at-homeness’ entails coordinated and mutually reinforcing rituals. It is well known that themes of closeness vs. distance are often associated with varying levels of well being and functionality in relationships. As a general trend it can be stated that closeness brings benefits for the relationships and distance is rather detrimental (Arriago et al., 2004). As noted earlier, these aspects may have their parallels in negative and positive forms of dyadic coping as well. Withdrawal, for example in form of alternating use of the common spaces, was a coping response to relationship stress in couples; from an STM perspective, these behaviors may be regarded as lack of support for the stressed partner, or even neglecting her needs. In contrast, joint shaping of the home (under the code ‘striving for at-homeness’) can be easily acknowledged as a positive, common dyadic coping act.

However, closer inspection of the variations of these main themes shows that opposite tendencies may be also found in both main themes. Conscious distancing may be an adequate and coordinated response to illness symptoms, although it still can cause adverse relationship experiences too. Couples also experienced alternating use of the spaces as sign of their well-coordinated coping with everyday challenges. In a similar way, joint strivings for at-homeness had their complex, sometimes even ambivalent character too. Interviewees spontaneously gave account of the risks of overprotection and delegated dyadic coping on behalf of the healthy partner. These ambivalent aspects of both ‘distancing’ and ‘joint strivings’ can be better understood when we consider that distancing may help overview, clarity and autonomy while closeness may eventually involve control and coercion (Kanat-Maymon et al., 2016). Therefore, our data suggest that there are complex, multifaceted interrelations between strategies of spatial behavior and dyadic coping processes. The inclusion of spatial aspects of dyadic coping seems especially important if we consider the results of Helgeson et al. (2017) who found that implicit ways of communal coping in the partner (e.g., in forms of we-talk) were especially beneficial for diabetes patients. Since much of spatial behavior is implicit in nature, we may assume that it conveys powerful messages about the relational meaning of the actual dyadic coping efforts.

#### Theoretical Outlook on Dyadic Coping Research

In sum, this research expands prior work on STM in the context of chronic disease by illustrating the profound role of the family home as a mediator. Recent research and commentary on the role of place in health (e.g., Corburn, 2017; Lovell et al., 2017) has tended to focus on higher, macro and meso levels of place, including health promoting support offered within one’s community of residence, or the impact of local and national policies on physical or mental health. Our results suggest that for many individuals, aspects of the family home, including features, distribution of space, and temporarily or long-term sharing with coresidential relatives, are simultaneously the background for and an aspect of dyadic coping in illness. When a family member is chronically ill, family functioning is challenged (Ryan et al., 2012) and the family’s satisfaction with the home environment can be linked to the fact if family members participate or are considered in the home design (Coulombe et al., 2016). Developing or worsening illness might be accompanied by increasing importance of the home environment with decreasing impact of macro and meso environments.

Bodenmann (2008) suggested disease specific patterns in coping exist. While distinguishing between coping for epilepsy versus chronic back pain was not a stated goal of this research, we respectfully suggest based on these findings, that use of the home space as a mediator of coping and self-regulatory activities was demonstrated throughout the sample. It might be that the explicit intent of home space shared or separate use varies based on condition, as suggested by one partner of a participant with epilepsy who perceived the home as a safer environment in the instance of a seizure, but it is possible there are factors at a higher level of abstraction, including safety or security, that are perceived consistently across various illness or disease states. Clearly, further research is needed to focus on the role of the home in dyadic coping within specific illness or disease states. While we did not formulate a comprehensive theoretical account on these phenomena, the results presented here may inspire a new line of investigation in order to develop a novel theory.

### Limitations

As with any individual interview research, data are subject to deliberate or inadvertent inaccuracies, although within the constructivist-interpretative research paradigm, we embrace data that reflect how individuals experience, interpret and chose to communicate reflect the reality(ies) of interest. Another limitation is presented by our use of the Emotional Map of the Home method, which incorporated an exploratory dimension to the research design over and above the exploration related to the research question. This framework had clear advantages in allowing us to gather data that were particularly thoughtful and nuanced because the process encouraged participants to provide a layer of interpretive reflection that enhanced and increased authenticity of simple descriptive examples. However, there were associated disadvantages in that we did not capture details of the illness experience that were not elicited through this method. Further research in areas related to environmental psychology such as proxemics and home safety perceptions and practices, and investigation of other types of chronic illness, are indicated to improve understanding of dyadic coping in chronic illness. That said, we believe our sample for this study was large and diverse enough to provide ample rich data to address the research purpose and facilitate initial theory development.

### Implications

These findings related to chronic illness, dyadic coping, and the family home have implications that researchers should explore in other contexts. Our findings related to the role of the family home in dyadic coping in the context of chronic illness are of potential importance for other domains of investigation. The role of home environment in the development of dyadic coping patterns can be studied in non-clinical samples like in relationships of emerging adults who seek to establish their adult life both in terms of the environmental circumstances and the basic rules (“relationship contract”; Sager, 1976; or “couple’s pact,” Cigoli and Scabini, 2006) of their long-term bond. Moreover, family home may be an important part of dyadic coping processes of families with special life situation other than chronic illness, such as families struggling with financial strains and challenges. Increases are anticipated in the proportion of older adults throughout the world, and many will experience some type of age-related disability or disease (United Nations Department of Economic and Social Affairs Population Division, 2015). Given population age trends, the previously discussed trend toward provision of an increasing number and scope of medical services in by carers in homes rather than institutions, and many individuals’ expressed preference for aging in place, improved understanding of the role the home plays in relationships, dyadic coping, and illness is an issue of ongoing importance. Consequently, our environmental psychologically informed approach toward relationships may be applied in professional trainings, patient education programs (c.f., Riley et al., 2001; Glasgow and Emmons, 2007) and may broaden the scope of health promotion in general. As an example, couple therapy and couple relationship enhancement programs focusing on dyadic coping strategies of the couples (e.g., CCET, Bodenmann and Shantinath, 2004; and TOGETHER, Falconier, 2015) may benefit from the spatial aspects of dyadic coping described here by making couples more aware of the spatial aspects of their behavior and from the qualitative data assessment methodology that we used here, that is, the Emotional Map of the Home Interview protocol that was intended not only for research but also for counseling purposes.

## Conclusion

In our analysis, we have demonstrated several key processes of how individuals living with chronic illness and close others implicitly or explicitly use their home environment in the process of coping with life conditions related to illness. Relationship science researchers and practitioners should address whether families living with a chronic illness understand how home–environment transactions can and do bear significance for their coping capacities, and, finally, to their emotional, relational and physical health. Our results may also contribute to more elaborated and complex theoretical considerations on self-regulation and dyadic coping processes. Living with chronic illness in the family home – whether as patient or a close relative – challenges dyadic coping skills and strategies at the highest level but may also discover hidden resources and possibilities. Our descriptions of transactive relationships between the partners, their relationship processes including dyadic coping efforts and the spatial-temporal context around them provide rich examples for both challenging and empowering aspects of the life situation of chronic illness.

## Data Availability

The raw data for this study (interview transcripts in Hungarian) supporting the conclusions of this manuscript will be made available by the authors, without undue reservation, to any qualified researcher.

## Ethics Statement

This study was carried out in accordance with the recommendations of the Codex of Ethics of Scientific Knowledge of the Hungarian Academy of Sciences, approved by the Medical Research Council (a board of the Hungarian Ministry of Human Capacities) with written informed consent from all subjects. All subjects gave written informed consent in accordance with the Declaration of Helsinki. The protocol was approved by the Medical Research Council.

## Author Contributions

VS and AD designed the study. VS carried out the research. VS and TM conducted data analysis. All authors contributed to the presented interpretation of findings. VS, TM, and SC wrote sections of the manuscript and read and all authors approved the final version.

## Conflict of Interest Statement

The authors declare that the research was conducted in the absence of any commercial or financial relationships that could be construed as a potential conflict of interest.

## Footnotes

1 http://dx.doi.org/10.3402/qhw.v9.23677

## Appendix: Full Code Tree

**Table:**

Code no.	Selective, axial and open codes
1.	Stress communication through the home space
1.1.	Direct stress communication is lacking – stress appears indirectly through space use
1.1.1	Tension cannot appear during the time spent together
1.1.2	There is no suffering in chronic illness
1.1.3	Only one of the partners needs conversation – thus it fails
1.1.4	Partners take turns when using home spaces
1.2.	Open disagreement appears in use of space
1.2.1	Disagreements on oppositely used spaces
1.2.2	Direct quarreling followed by withdrawal
1.2.3	Quarrels occur while in bed together, sexual relationship fails
1.3.	Differences in partners’ priorities for space use cause conflicts
1.3.1	Continuing conflict when one partner defers to the other’s priorities
1.3.2	Distribution of space as a proxy for feelings of value
1.3.3	Control fight for the possession of the territories
2.	Coping by spatial separation
2.1.	Conscious distancing in coping with the symptoms
2.1.1	Alleviation of symptoms through daily rituals of retiring
2.1.2	Wife’s duty to care for sick child distances partners
2.1.3	Sleeping separately helps alleviate symptoms
2.1.4	The healthy partner has “nothing to do” with the coping with the other’s illness
2.2.	Distancing in the coping with relationship stress
2.2.1	Rituals of withdrawal inside the shared room
2.2.2	Partners avoid each other in the bedroom – lack of sexual life
2.2.3	Rituals of withdrawal to the next room
2.2.4	Childhood experiences informing rituals of withdrawal
2.2.5	The ritual of creating an alternative home
2.3.	Mutually reinforcing processes of distancing
2.3.1	Growing distance in the pursuit of a home related goal
2.3.2	A recovery supporting home environment creates distance in the relationship
2.3.3	The way of parental care creates distance
3.	Coping by joint striving for at-homeness
3.1.	One partner takes care of the other in the absence of stress communication
3.1.1	The healthy partner provides security for the ill partner in the space of the home
3.1.2	The ill partner’s loss of control more profoundly affects her partner
3.1.3	The healthy partner initiates a shared ritual to alleviate the other’s symptoms
3.2.	The risk of taking over home duties
3.2.1	The ill partner considers insufficient the duties taken over
3.2.2	The partner’s help is inefficient/other help would be needed
3.2.3	The takeover of duties is followed by blaming
3.2.4	The healthy partner gets tired of taking over duties and shows symptoms
3.3.	The common use of the home space supports coping with stress
3.3.1	The common actions around the children’s needs provide a pleasant feeling
3.3.2	The couple’s common actions in the home space provide security and healing
3.3.3	Being together at home compensates for the changes in relationships
3.4.	The joint shaping of the home space
3.4.1	Changing use of the home space can provide hope
3.4.2	The furniture collaboratively created by the couple can support coping with the symptoms
